# Pak1 dysregulates pyruvate metabolism in PDAC cells by exerting a phosphorylation-mediated regulatory effect on PDHA1

**DOI:** 10.1016/j.jbc.2025.108409

**Published:** 2025-03-14

**Authors:** Sowmiya Murugan, Srikanth Swamy Swaroop B, Prarthana Gopinath, Roshni Saravanan, Sandhya Sundaram, Gouthaman Shanmugasundaram, Ganesh Venkatraman, Suresh Kumar Rayala

**Affiliations:** 1 Molecular Oncology Laboratory, Department of Biotechnology, Indian Institute of Technology Madras, Chennai, Tamil Nadu, India; 2 Department of Human Genetics, Sri Ramachandra Faculty of Biomedical Sciences & Technology, Sri Ramachandra Institute of Higher Education and Research, Chennai, Tamil Nadu, India; 3 Department of Pathology, Sri Ramachandra Medical College, Sri Ramachandra Institute of Higher Education and Research, Chennai, Tamil Nadu, India; 4 Department of Surgical Oncology, Apollo Speciality Hospitals, Chennai, Tamil Nadu, India; 5 Department of Bio-Medical Sciences, School of Bio Sciences & Technology, Vellore Institute of Technology, Vellore, Tamil Nadu, India

**Keywords:** Pak1, pyruvate dehydrogenase, Warburg effect, PDAC

## Abstract

Pancreatic ductal adenocarcinoma (PDAC) is a highly aggressive form of pancreatic cancer with the worst prognosis. Treating PDAC poses significant challenges, as tumor cells adapt metabolic alterations to thrive in the hypoxic environment created by desmoplasia surrounding the tumor cells. p21-activated kinase (Pak1), a serine-threonine kinase is found to be upregulated in many solid tumors and promotes tumor progression *via* diverse signaling pathways. In this study, we focused on exploring the role of Pak1 in mediating tumor cell metabolism. Deletion of the *Pak1* gene reduced the tumorigenic potential of PDAC cells. Also, Pak1 regulated both glycolysis and mitochondrial respiration in PDAC cells, contributing to the Warburg phenomenon. Untargeted metabolomic analysis revealed that Pak1 was strongly associated with pyruvate metabolism. Interestingly, we found that Pak1 interacted and phosphorylated pyruvate dehydrogenase E1α (PDHA1) at serine 152. This phosphorylation negatively regulates PDHA1 activity, implying the direct regulatory role of Pak1 in pyruvate metabolism. Moreover, deleting the *Pak1* gene altered the expression and activity of PDHA1 and LDHA, as both are involved in regulating the direction of pyruvate flux inside the cells. Our study demonstrated that Pak1 plays a significant role in PDAC metabolism and Warburg effect, partly by phosphorylating PDHA1.

Pancreatic ductal adenocarcinoma (PDAC) which accounts for over 95% of pancreatic cancer cases, has been identified as one of the most devastating and lethal malignancies. With an alarmingly low 5-year survival rate of less than 5%, pancreatic cancer has become the fourth leading cause of cancer-related deaths, mainly due to ineffective clinical treatment options and late diagnosis (1, 2). The aggressive nature of PDAC is characterized by a strong desmoplastic reaction surrounding the tumor cells, creating a hypoxic environment and nutrient depletion (3). To sustain in this metabolically challenging environment, the tumor cells adapt by reprogramming their metabolic pathways to support tumor proliferation and survival. PDAC cells exhibit high glucose uptake, increased glycolysis and reduced mitochondrial respiration—termed as Warburg effect (4). Tumor cells attain these metabolic alterations by exerting increased dependency on certain oncogenes, primarily mediated by oncogenic KRAS, mutated in approximately 90% of PDAC cases (5). The activation of diverse signaling pathways by oncogenic KRAS facilitates tumor cell proliferation. One notable downstream effector of KRAS signaling pathways is Pak1, or p21-activated kinase 1, which belongs to the family of serine/threonine kinases. Elevated expression of Pak1 is often observed in tumors, particularly in KRAS-driven tumors. Pak1 is vastly recognized for its involvement in integrating various signaling pathways associated with cell proliferation, survival, cell cycle, and apoptosis through phosphorylation of relevant substrates (6). Moreover, Pak1 serves as a scaffold, exhibiting kinase-independent activity by facilitating interactions among proteins (7, 8). Previous reports highlight Pak1's intrinsic involvement in driving pancreatic tumorigenesis and its role in acquiring gemcitabine resistance (9). Apart from the aforementioned roles, Pak1 has also been identified as a metabolic regulator in facilitating whole-body glucose homeostasis by regulating glucose uptake in skeletal muscle, pancreatic β-cell insulin release, and glucagon-like peptide secretion in the intestine. Pak1 inhibits phosphoglyceromutase activity by phosphorylation redirecting the metabolism toward the pentose phosphate pathway in neutrophils (10). Moreover, Pak1 phosphorylates phosphoglucomutase, augmenting its enzymatic activity, which preferentially favors the conversion of glucose-1-phosphate to glucose-6-phosphate, a glycolytic intermediate (11). Many cases of PDAC show increased expression of the transcription factor c-Myc, which promotes higher expression of glycolytic genes and has been identified as a downstream target of Pak1 (12, 13). Although numerous scientific reports link Pak1 to tumorigenesis, there is a paucity of research exploring Pak1’s role in regulating tumor cell metabolism. This study focuses on Pak1's specific impact on metabolic pathways within tumor cells, which may provide valuable insights into the metabolic adaptations driving PDAC tumorigenesis.

## Results

### Elevated Pak1 expression fuels the oncogenic traits of PDAC cells

**Figure 1:**
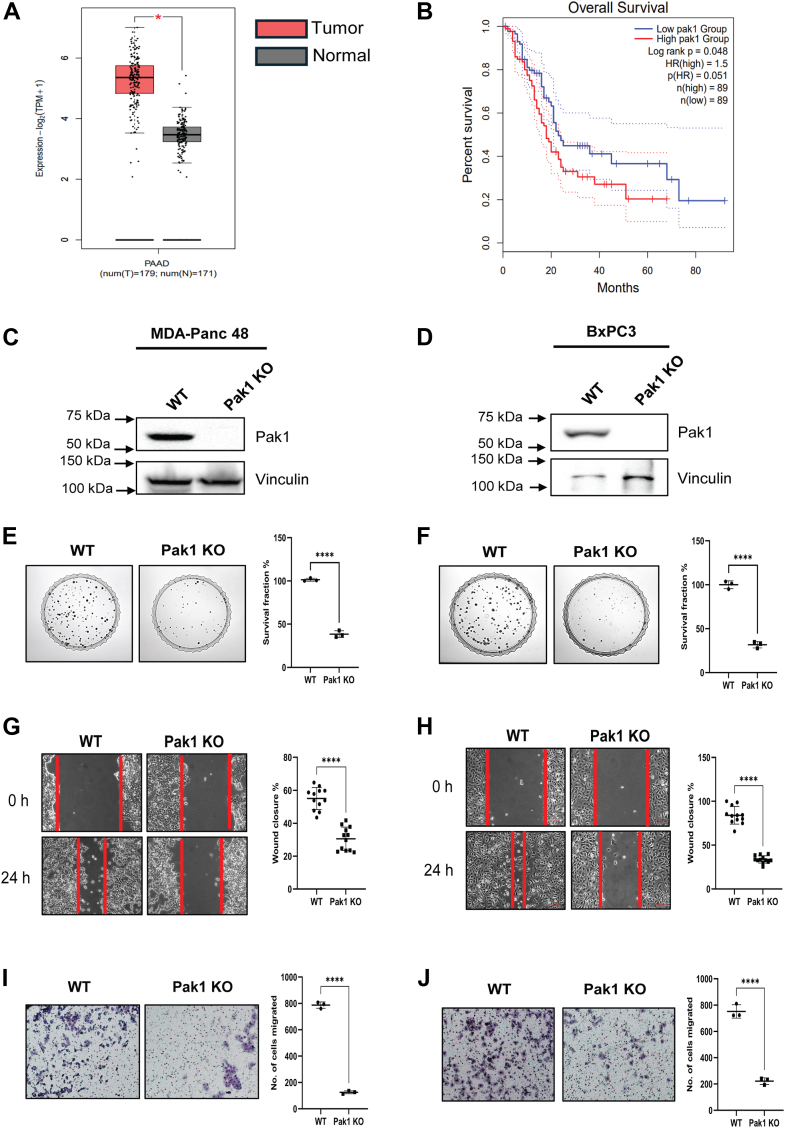
**Pak1 is upregulated in PDAC tissues and Pak1-specific gene deletion reduces the tumorigenic potential of PDAC cell lines.***A*, the gene expression level of Pak1 in normal and PDAC tissues was derived from the GEPIA2 analysis tool. *B*, the overall survival analysis of PDAC patients with high and low Pak1 expression was obtained using the GEPIA2 analysis tool. *C* and *D*, Western blot analysis confirmed the stable Pak1 KO clone generated in the MDA-Panc 48 and BxPC3 cell lines. The tumor-forming ability of WT and Pak1 KO cells was assessed by performing. *E* and *F*, colony formation assay. *G* and *H*, wound healing assay. *I* and *J*, transwell migration assay. Each value represented is mean ± SD from three biological replicates. *(∗p < 0.05, ∗∗p < 0.005, ∗∗∗p < 0.0005, and ∗∗∗∗p < 0.0001 compared with WT cells).* Pak1, p21-activated kinase; PDAC, pancreatic ductal adenocarcinoma.

To elucidate the abundance of Pak1 in PDAC, we first analyzed the *Pak1* gene expression level in PDAC tissues and normal tissues using GEPIA2, an interactive online analysis tool (Fig. 1*A*). The data showed that *Pak1* is highly expressed in pancreatic tumor tissue (T = 179) when compared to the normal pancreatic tissue (N = 171). Survival analysis showed that the overall survival rate of PDAC patients with high *Pak1* gene expression is significantly lower than the group with low *Pak1* gene expression (Fig. 1*B*). We also performed immunohistochemical (IHC) staining of Pak1 in a human tissue microarray (TMA) comprising PDAC tissues (n = 40) and adjacent normal pancreatic tissue (n = 40). Consistent with the GEPIA2 findings, the IHC data showed that Pak1 expression is significantly upregulated in the PDAC tissues with a mean q-score of 147.8, when compared to the normal tissue with a mean q-score of 77.70 (Supplementary information, S1).

We generated the stable Pak1 KO in the PDAC cell lines, MDA-Panc 48, and BxPC3 using CRISPR-Cas9 KO plasmid (specific to *Pak1*) as a model system to study the role of Pak1 in PDAC tumorigenesis. Western blot analysis confirmed the absence of Pak1 expression in the clone generated in both the cell lines, MDA-Panc 48, and BxPC3 (Fig. 1, *C* and *D*). To assess the tumorigenic property of Pak1 in PDAC cells, we performed the clonogenic cell survival assay, the wound healing assay, and the transwell migration assay in WT and Pak1 KO cell lines generated. In the clonogenic cell survival assay, the number of colonies formed by Pak1 KO clone drastically reduced as compared to the WT, in both MDA-Panc 48 and BxPC3 cells. The survival fraction of PDAC cells was significantly reduced upon deleting the Pak1 gene (Fig. 1, *E* and *F*). In MDA-Panc 48 Pak1 KO cell line, there is a drastic reduction in the percentage of wound closure as compared to the WT cells (Fig. 1*G*). Similarly, the BxPC3 Pak1 KO cell line showed impaired wound healing capacity than the WT cells (Fig. 1*H*). Furthermore, the transwell migration assay data also revealed a significant decrease in the number of cells migrated in Pak1 KO cell line compared to the WT, in both MDA-Panc 48 and BxPC3 cells (Fig. 1, *I* and *J*). Collectively, these results showed the predominant role of Pak1 in promoting tumorigenesis by affecting the survival and migratory potential of PDAC cells.

### Pak1 drives aerobic glycolysis to sustain metabolic demands of PDAC cells

**Figure 2:**
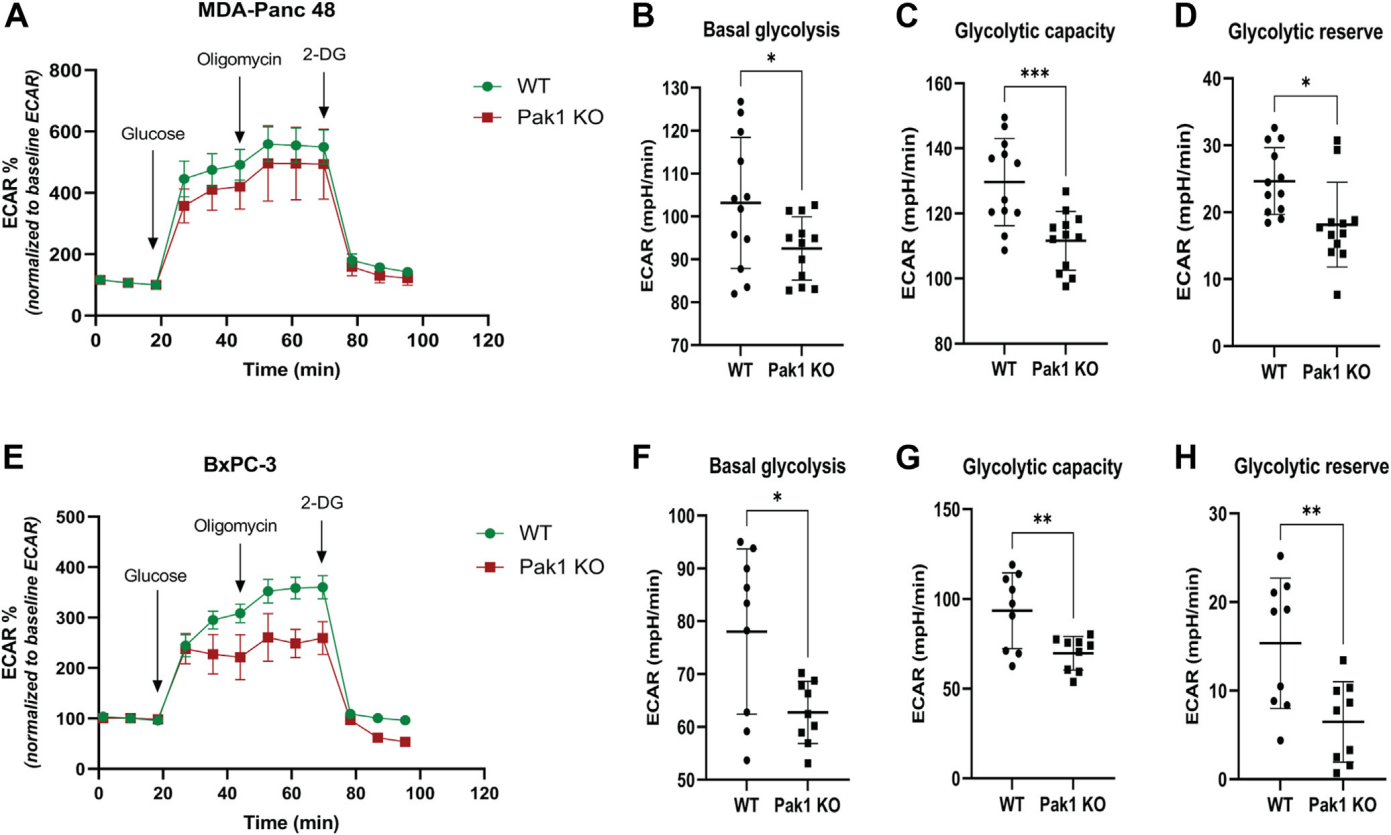
**Pak1 impacts glycolysis in PDAC cells.** Assessment of the glycolytic activity of WT and Pak1 KO clone by using Seahorse extracellular flux (XF24) analyser: Graphical representation of changes in the extracellular acidification rate (ECAR, mpH/min) upon the addition of glucose, oligomycin, and 2-deoxy glucose in MDA-Panc 48 (*A*) and BxPC3 (*E*). The ECAR values of glycolytic parameters such as basal glycolysis, glycolytic capacity, and glycolytic reserve capacity were calculated for WT and Pak1 KO clones: MDA-Panc 48 (*B–D*) and BxPC3 (*F*–*H*). Each value represented is mean ± SD derived from at least two independent experiments. *(∗p < 0.05, ∗∗p < 0.005, ∗∗∗p < 0.0005, and ∗∗∗∗p < 0.0001 compared to the WT).* Pak1, p21-activated kinase; PDAC, pancreatic ductal adenocarcinoma.

We aimed to investigate the role of Pak1 in regulating the energy demands of continuously proliferating tumor cells. In general, the tumor cells adapt metabolic changes to fuel uncontrolled cell proliferation, by increasing glycolysis and reducing mitochondrial respiration. To gain insights into the Pak1 role in glycolysis, the extracellular flux analyzer was used to measure the changes in metabolic activity of the cells. The WT and Pak1 KO cells of both MDA-Panc 48 and BxPC3 were subjected to the glycolysis stress test to measure the glycolytic activity of the cells. Figure 2*A* displays the graphical representation of the change in extracellular acidification rate (ECAR) over time for both WT and Pak1 KO cells in MDA-Panc 48, while Figure 2*E* represents the same for BxPC3 cells. The results showed that the Pak1 KO cells displayed a significant reduction in ECAR values in both cell lines implying reduced glycolytic activity. The glycolytic parameters such as basal glycolysis, maximal glycolytic capacity, and glycolytic reserve were calculated in both WT and Pak1 KO cells. In the MDA-Panc 48 cell line, Pak1 gene deletion resulted in a reduced basal glycolysis level relative to the WT cells. Also, the maximal glycolytic capacity significantly decreases in the Pak1 KO cell line. Depleting the Pak1 gene also led to a diminished glycolytic reserve as compared to WT (Fig. 2, *B*–*D*). Likewise, the BxPC3 cell line also demonstrated reduced basal glycolysis and maximal glycolysis levels in Pak1 KO cells as compared to the WT cells. Only half of the glycolytic reserve capacity was retained in BxPC3 Pak1 KO cell line as compared to the WT (Fig. 2, *F*–*H*). The results from the glycolysis stress test for both cell lines, MDA-Panc 48 and BxPC3 provide indisputable evidence of the necessity of Pak1 in maintaining higher glycolytic activity in PDAC cells.

**Figure 3:**
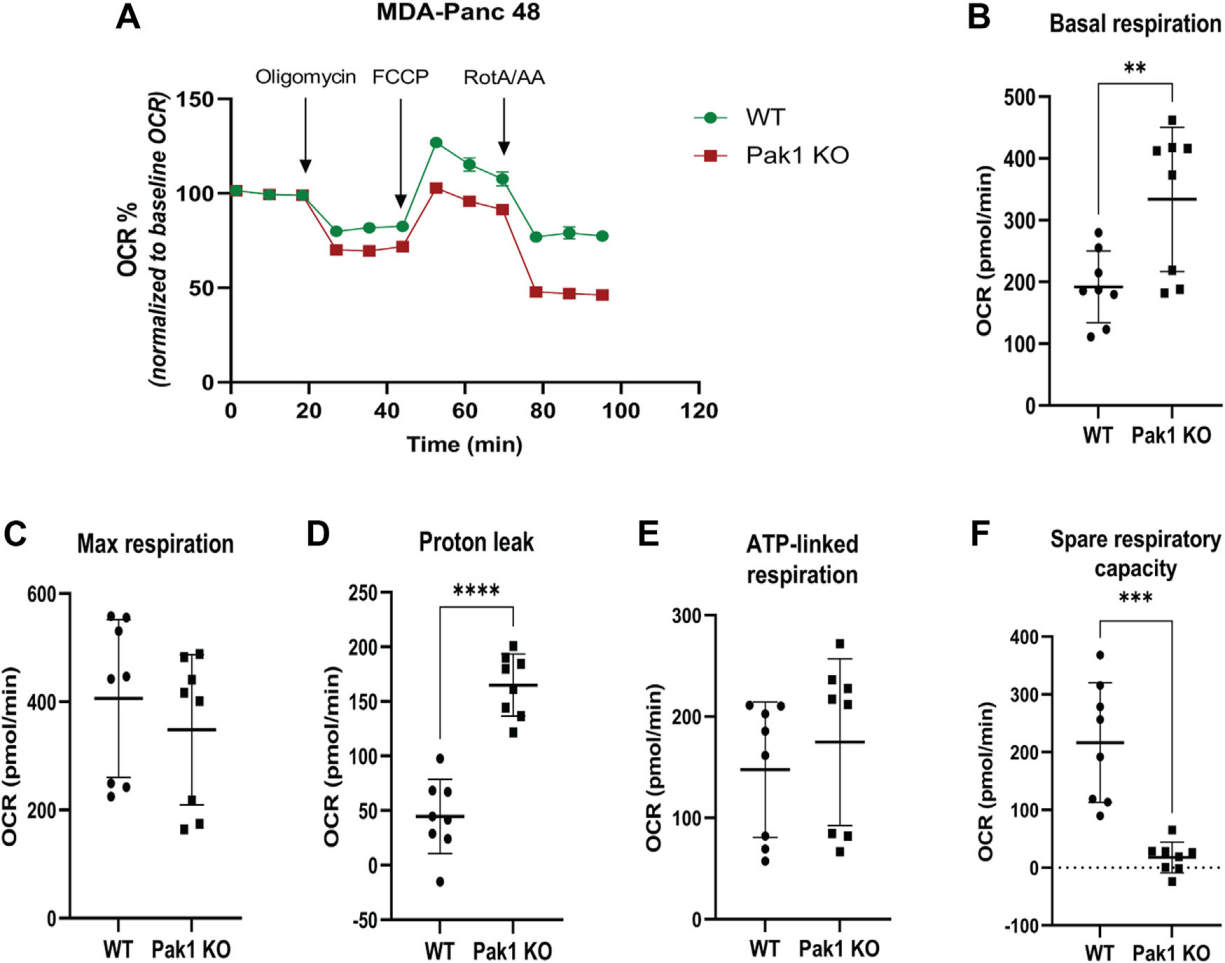
**Pak1 alters mitochondrial respiration in PDAC cells.** Assessment of the mitochondrial capacity of WT and Pak1 KO clone by using Seahorse extracellular flux (XF24) analyzer. *A*, graphical representation of change in the oxygen consumption rate (OCR, pmol/min) upon addition of oligomycin, FCCP and rotenone A/antimycin in MDA-Panc 48 WT and Pak1 KO clone. *B*–*F*, the OCR values for mito parameters such as basal, maximum respiration, ATP-linked respiration, proton leak, and spare respiratory capacity in both WT and Pak1 KO clones in MDA-Panc 48. Data represented as mean ± SD derived from two independent experiments. *(∗p < 0.05, ∗∗p < 0.005, ∗∗∗p < 0.0005, and ∗∗∗∗p < 0.0001 compared to the WT)*. FCCP, carbonyl cyanide-4 (trifluoromethoxy) phenylhydrazone; Pak1, p21-activated kinase; PDAC, pancreatic ductal adenocarcinoma.

Furthermore, we studied the effect of Pak1 in the regulation of mitochondrial respiration by performing the Mito stress test in WT and Pak1 KO cells by measuring the oxygen consumption rate (OCR). The change in OCR value upon adding the mitochondrial inhibitors was used to compute the key parameters relevant to the mitochondrial function. A graphical representation of OCR response over time is shown in Figure 3*A*, for MDA-Panc 48 WT and Pak1 KO cells. The baseline OCR was measured to determine the respiration rate in the basal state. The basal respiration of Pak1 KO cells is significantly higher than the WT (Fig. 3*B*). There is no significant improvement in maximal respiration and ATP-linked respiration in the Pak1 KO clones (Fig. 3, *C* and *E*). A significant increase in OCR due to proton leak was observed in the Pak1 KO clone as compared to the WT (Fig. 3*D*). Increased proton leak without significant change in ATP-linked respiration in Pak1 KO cells shows disrupted coupling of proton movement with oxidative phosphorylation, indicating reduced mitochondrial efficiency. Also, the spare respiratory capacity of the Pak1 KO cell line was significantly reduced, impairing the ability of the cell to meet the energy demand under stress conditions (Fig. 3*F*). The BxPC3 Pak1 KO cells showed poor response to the mitochondrial inhibitors in Mito stress test (*data not shown*). Though the Pak1 KO clone increased basal respiration level, the overall mitochondrial function was not improved. Taken together, the results show that Pak1 has a prominent role in regulating the metabolic pathways of the PDAC cells.

### Overall metabolic changes imparted by Pak1 in PDAC cells

**Figure 4:**
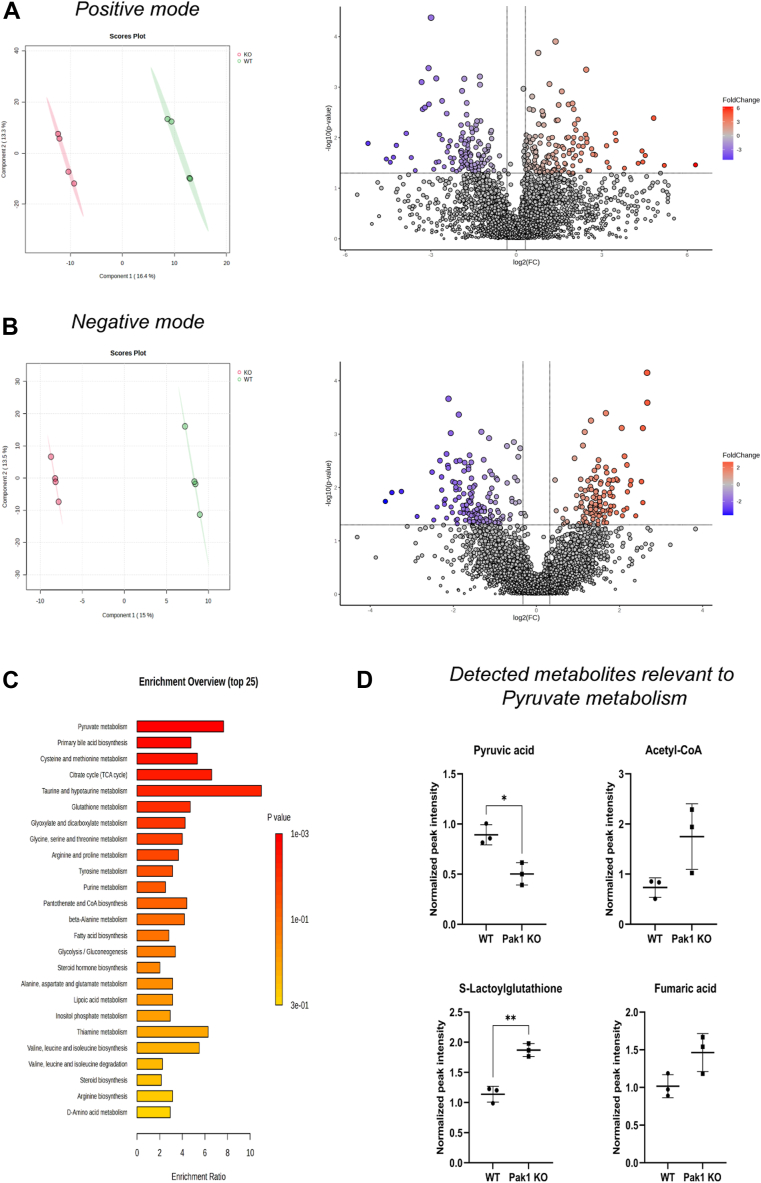
**LC-MS/MS data visualization and enrichment analysis of DAMs in MDA-Panc 48 cells.***A* and *B*, *left panel*: PLS-DA plot of WT and Pak1 KO in positive mode (4a, *left panel*) and negative mode (4b, *left panel*). *A* and *B*, *right panel:* volcano plot illustrates the distribution of metabolite features identified in positive (4a, *right panel*) and negative mode (4b, *right panel*). The metabolite features that are differentially altered in Pak1 KO with a *p value* ≤ 0.05 were highlighted in *red* (upregulated) and *blue* (downregulated). *C*, representation of enrichment analysis was performed using the KEGG metabolite set library as a reference with the list of differentially abundant metabolites (DAMs) identified in Pak1 KO cells (0.75 ≤ FC ≥ 1.3). *D*, box plot representation of the normalized peak intensity value of metabolites identified in pyruvate metabolism in WT and Pak1 KO cells. *(∗p < 0.05, ∗∗p < 0.005, ∗∗∗p < 0.0005, and ∗∗∗∗p < 0.0001 compared to the WT).* DAM, differentially altered metabolite; KEGG, Kyoto Encyclopedia of Genes and Genomes; FC, fold change; Pak1, p21-activated kinase; PLS, partial least squares; PLS-DA, PLS discriminant analysis.

Global metabolic profiling using LC-MS/MS analysis identified the changes in the metabolic profile in PDAC cells implicated upon Pak1 gene deletion. Collectively, 2399 aligned peak features in positive mode and 1384 aligned peak features in negative mode were identified based on the retention time, isotope ratio, accurate *m/z* value, and matching mass spectrometry (MS) spectra with the spectral library data used. The peak features lacking the MS2 level assignment were assigned to the precursor level by matching their respective *m/z* value. In positive mode, 2109 features were assigned at the precursor level (MS1) and 290 features were detected at the MS2 level. Out of 1384 features detected in negative mode, 1293 were detected at the precursor level (MS1) and 91 at the MS2 level. With the help of partial least squares discriminant analysis, the clustering pattern among the biological replicates in the group was studied. As shown in Figure 4, *A* and *B* (*left panel)*, we observed a distinct separation between the groups (WT and KO) and tight clustering among its biological replicates, in both positive and negative modes. The differentially altered metabolite (DAM) features were identified by performing the fold-change (FC) analysis. The volcano plot depicting the DAMs features with the FC threshold of 1.3 (0.75 ≥ FC ≥ 1.3) with *p value* ≤ 0.05 for both modes is shown in Figure 4, *A* and *B* (*right panel*). The list of DAMs is given in the Supplementary information (S2). Subsequently, the identified DAMs were used for enrichment analysis with Kyoto Encyclopedia of Genes and Genomes as the reference metabolite set, to gain insight into the metabolic pathways altered upon Pak1 gene deletion. The enrichment analysis identified pyruvate metabolism to be the top significant pathway altered by Pak1 (Fig. 4*C*). Emphasizing the pivotal role of pyruvate metabolism, the metabolites associated with this pathway that exhibit varied levels of expression are pyruvate, acetyl-CoA, fumaric acid, and S-lactoylglutathione. The normalized peak intensity values of each of these metabolites are represented in Figure 4*D*. The normalized peak intensity value of pyruvate is significantly less in Pak1 KO cells as compared to the WT. Conversely, the normalized peak intensity value of acetyl-CoA increased in Pak1 KO cells compared to the WT. The metabolite fumaric acid (an intermediate in the tricarboxylic acid cycle) is increased in Pak1 KO cells as compared to WT. The normalized peak intensity value of S-lactoylglutathione, which is involved in methylglyoxal detoxification, is also increased in Pak1 KO cells as compared to WT. Both fumaric acid and S-lactoylglutathione are not part of primary pyruvate utilization. Global metabolomic analysis in WT and Pak1 KO cells shows that Pak1 regulates pyruvate metabolism, which serves as the nexus of the carbon metabolic pathway of the cells.

### Deciphering the link between Pak1 and pyruvate metabolism

We aimed to establish the regulatory link of Pak1 and pyruvate metabolism—a pathway of prime importance, which lies in the distinction between normal and cancer cells. In cells, pyruvate to acetyl-CoA conversion is mainly mediated by pyruvate dehydrogenase complex with an integral enzymatic component, pyruvate dehydrogenase E1α subunit (PDHA1). We observed changes in both pyruvate and acetyl-CoA levels, which led us to speculate that Pak1 might have a direct influence on the enzyme involved in this reaction. Second, it is well established that the activity of PDHA1 is regulated by reversible phosphorylation; we hypothesized PDHA1 to be a potential substrate of Pak1.

### Pak1 interacts with and phosphorylates PDHA1

**Figure 5:**
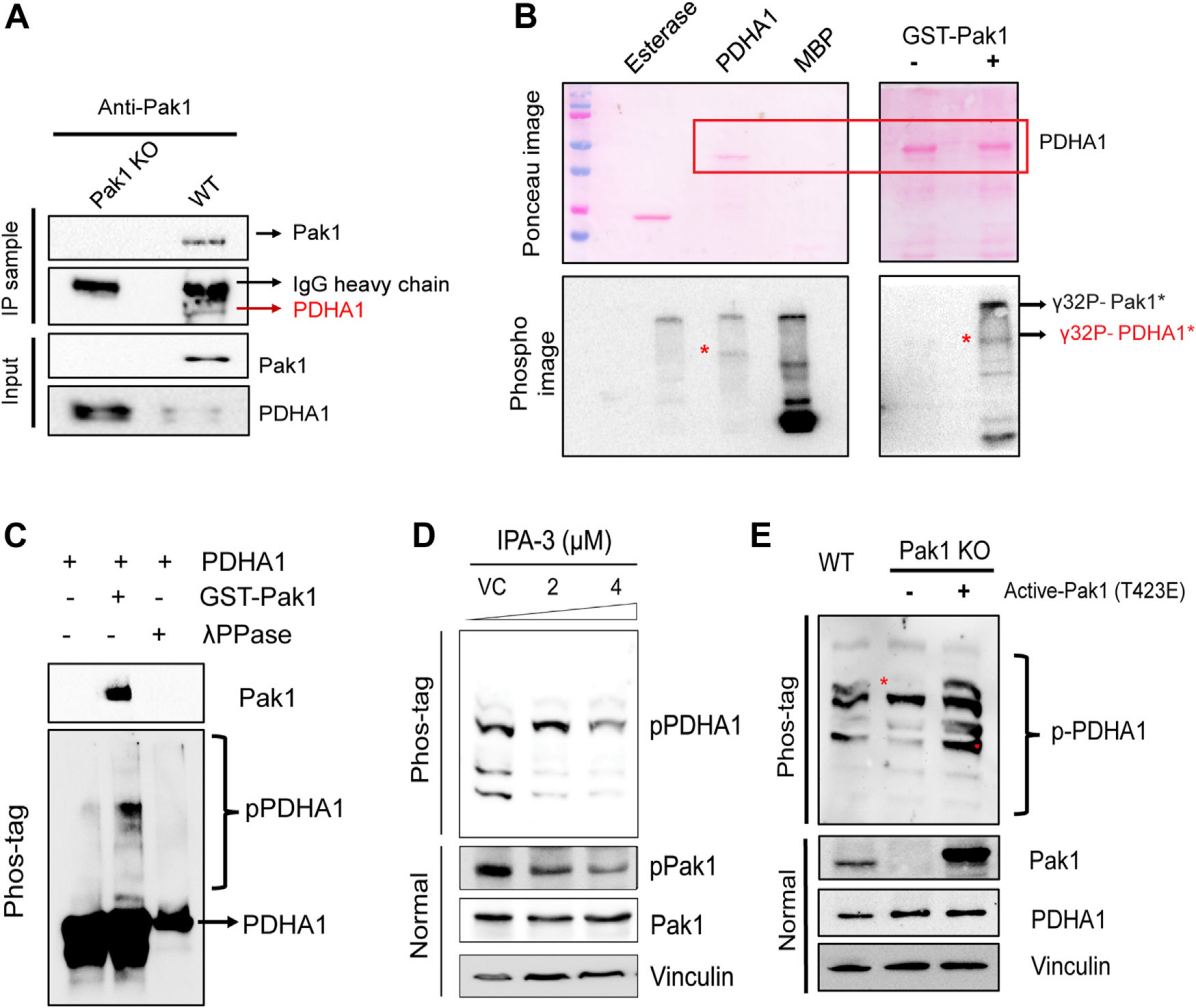
**Delineating the regulatory relationship of Pak1 on PDHA1.***A*, coimmunoprecipitation experiment showed PDHA1 interacts with Pak1 in MDA-Panc 48 cells under physiological conditions (MDA-Panc 48 Pak1 KO cell lysate was used as the control to detect nonspecific binding). *B*, *in vitro* kinase assay performed using γ^32^-ATP: (*B*, *left panel*) assay performed with PDHA1 (target protein), MBP (positive control), and esterase (negative control); (*B*, *right panel*) assay performed with and without Pak1; *C*, phosphorylation of PDHA1 in the presence of Pak1 observed in Phos-tag gel. *D*, change in the PDHA1 phosphorylated protein levels upon treating the MDA-Panc 48 cells with IPA-3 inhibitor at 2 and 4 μM. *E*, change in the pPDHA1 levels upon restoration of constitutively active Pak1 expression transiently. MBP, myelin basic protein; Pak1, p21-activated kinase; PDHA1, pyruvate dehydrogenase E1α subunit.

First, interaction of Pak1 with PDHA1 in PDAC cells was demonstrated using coimmunoprecipitation with WT and Pak1 KO lysate. Coimmunoprecipitation with Pak1-specific antibody detected PDHA1 in WT, but not in KO, confirming the interaction of Pak1 and PDHA1 in PDAC cells (Fig. 5*A*). To assess the phosphorylation of PDHA1 by Pak1, *in vitro* kinase assay was performed with Pak1 kinase and recombinant hPDHA1 as substrate with myelin basic protein as positive control and esterase as negative control. Appearance of γ-P^32^–labeled band at ∼42 kDa in PDHA1 lane showed that Pak1 phosphorylates PDHA1. Moreover, the kinase assay set without Pak1 confirmed that the detected PDHA1 band was primarily a result of Pak1 phosphorylating PDHA1, as opposed to Pak1 autophosphorylation (Fig. 5*B*). Furthermore, the phosphorylation of PDHA1 by Pak1 was confirmed by Phos-tag SDS-PAGE, where the phosphorylated form of the protein migrates slower than the unphosphorylated form, leading to a band shift. Separation of the phosphorylated bands on Phos-tag gel was observed only in the presence of Pak1, indicating that PDHA1 was phosphorylated by Pak1 (Fig. 5*C*). To expand our understanding of this phosphorylation event *in vivo*, MDA-Panc 48 cells were treated with Pak1 inhibitor, IPA-3 at 2 and 4 μM for 36 h. Inhibition of Pak1 by IPA-3 was confirmed by the decline in pPak1/Pak1 levels on Western blot. This pharmacological inhibition of Pak1 led to a notable reduction in the intensity of the retarded phosphorylated PDHA1 bands as observed on the Phos-tag SDS-PAGE. (Fig. 5*D*). When MDA-Panc 48 WT and Pak1 KO cells were subjected to Phos-tag analysis, it showed a significant reduction in the intensity of the phosphorylated PDHA1 band in Pak1 KO lysate. Transient overexpression of kinase-active Pak1-T423E plasmid in Pak1 KO cells, significantly increased the phosphorylated PDHA1 bands on Phos-tag blots, similar to the pPDHA1 bands observed in WT lysate (Fig. 5*E*). Collectively, these results provide substantial evidence that Pak1 interacts and phosphorylates PDHA1 in PDAC cells.

### Pak1 phosphorylates PDHA1 on serine 152

**Figure 6:**
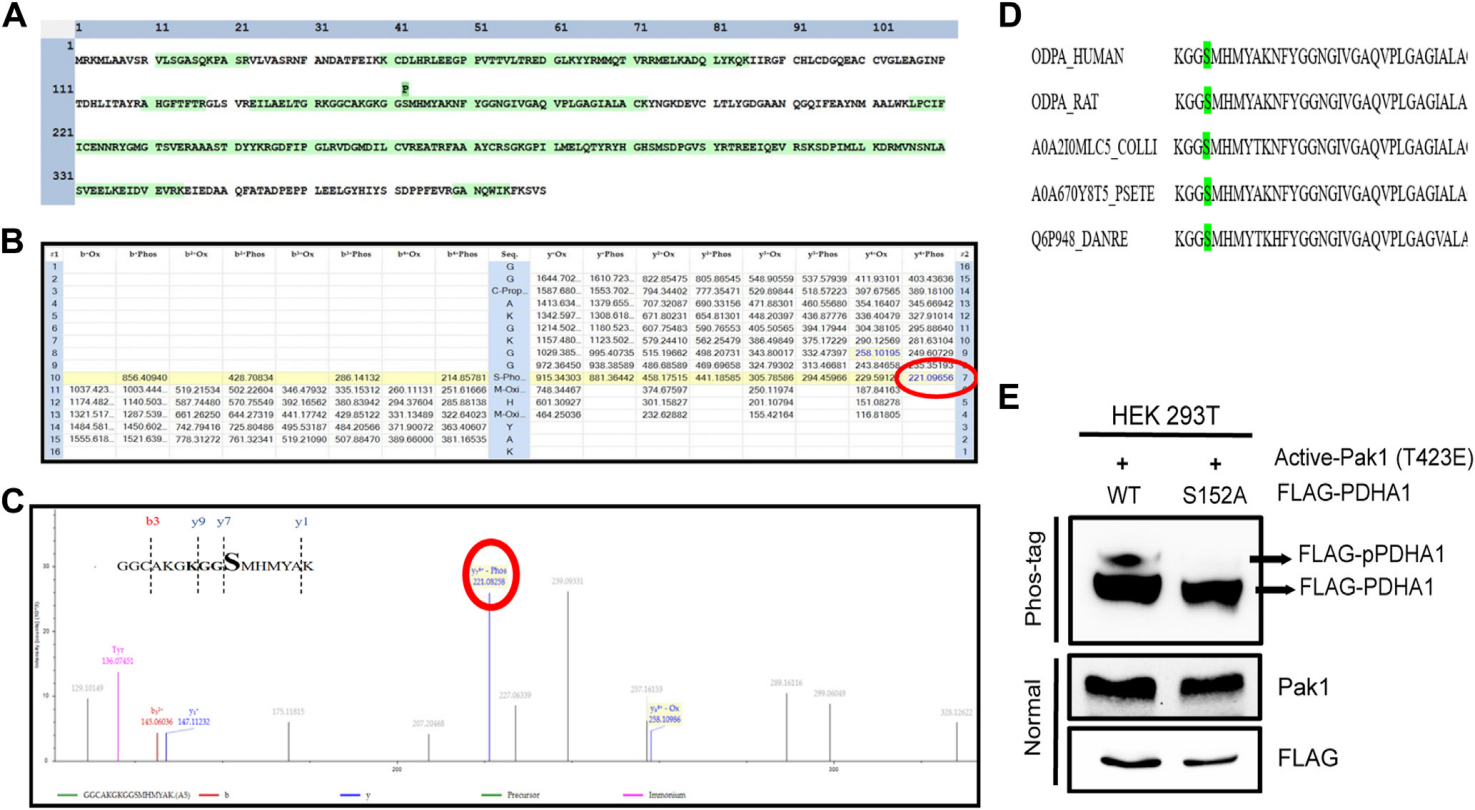
**Pak1 phosphorylates PDHA1 at Ser152 residue.***A*, graphical representation of sequence coverage in PDHA1 showing phosphorylation at S152. *B*, diagnostic ion table with the theoretical *m/z* values. Modifications are seen as a neutral loss on higher energy collisional dissociation fragmentation. *C*, mass spectra of the peptide GGCAKGKGGsMHMYAK. Inset shows the b and y ions identified from the peptide fragmentation and PAK1 consensus sequence (*bold*). *D*, ClustalW Multiple sequence alignment of PDHA1 from different species—human, rat, dove, snake, and zebrafish. The S152 (*green*) is conserved across species. *E*, Phos-tag immunoblot of PDHA1-WT and PDHA1-S152A cotransfected with PAK1 T423E. PAK1 overexpression and loading were confirmed on a parallel blot. Pak1, p21-activated kinase; PDHA1, pyruvate dehydrogenase E1α subunit.

*In vitro* kinase assay sample of PDHA1 with and without Pak1 analyzed by mass spectroscopy confirmed Pak1-specific phosphorylation at S152 with a site probability score of 100% (Fig. 6*A*). The modified to unmodified peptide spectral matches (PSMs) were 3/33 for S152, which was identified as unmodified PSM in control group, in the absence of Pak1. Phosphorylation on S152 was observed as neutral loss in higher energy collisional dissociation fragmentation, with theoretical *m/z* value of 221.09656 (Fig. 6*B*), matching with the experimental *m/z* 221.08258 (Fig. 6*C*). The identified phosphosite (GGCAKGKGGsMHMYAK) was also found to consist of Pak1 consensus sequence of R/KXXS/T. Serine at the 152 position in PDHA1 was found to be conserved among various species, signifying functional implication of the modification (Fig. 6*D*). Thus, S152 on PDHA1 was identified as the site of phosphorylation for Pak1. To confirm the phosphorylation at S152, alanine mutant (PDHA1 S152A) was generated and analyzed using Phos-tag SDS-PAGE. Elaborately, HEK293T cells were cotransfected with PDHA1 WT or PDHA1 S152A and Pak1 T423E plasmids. Retarded migration of phosphorylated PDHA1 was observed in PDHA1 WT, which was absent in PDHA1 S152A, denoting the S152-specific phosphorylation (Fig. 6*E*). Based on the experimental data presented thus far, it was inferred that Pak1 phosphorylated PDHA1 at S152.

### Pak1 negatively regulates the PDHA1 activity

**Figure 7:**
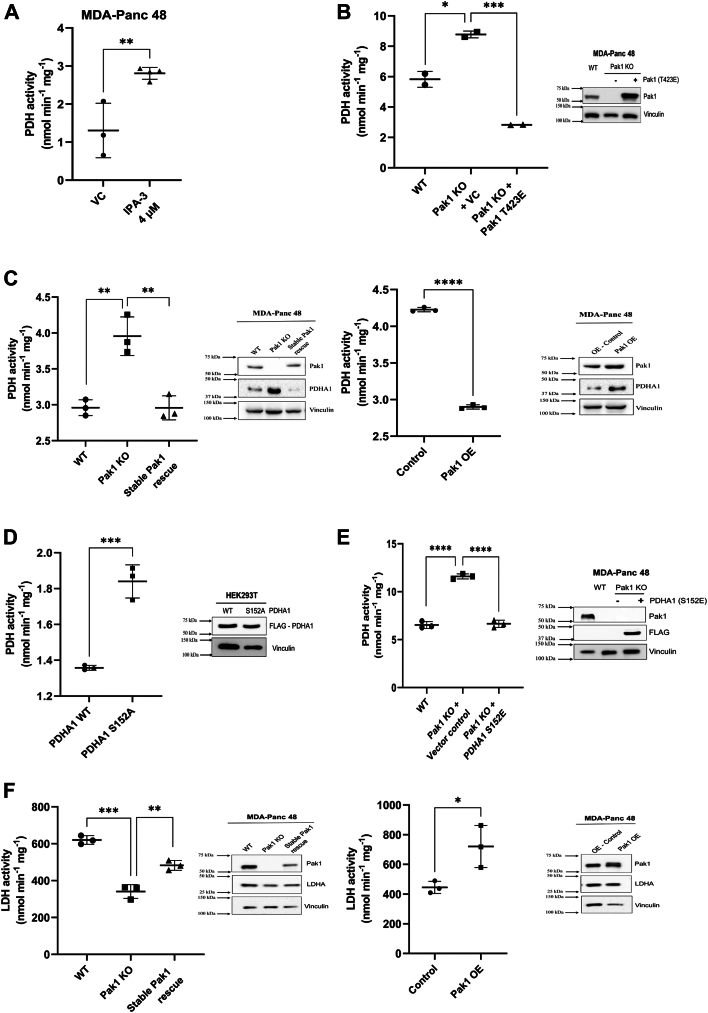
**Pak1 phosphorylating PDHA1 inhibits PDHA1 activity.***A*, MDA-Panc 48 cells treated with Pak1 inhibitor, IPA-3 (4 μM). *B*, transient expression of constitutively active Pak1 in MDA-Panc 48 cells Pak1 KO cells. *C*, PDHA1 activity in stable Pak1 clones: Pak1 KO, stable rescue, and Pak1 overexpression (Pak1 OE) cells. *D*, transient expression of FLAG-PDHA1 WT or phospho-null mutant (S152A) plasmid in HEK293T cells. *E*, transient expression of FLAG-PDHA1 WT or phosphor-mimic (S152E) plasmid in MDA-Panc 48 Pak1 KO cells. *F*, LDHA activity assay measured in stable Pak1 clones: Pak1 KO, Pak1 KI, and Pak1 OE cells. *Data represented as mean ± SEM (n = 3). ∗p < 0.05, ∗∗p < 0.005, ∗∗∗p < 0.0005, and ∗∗∗∗p < 0.0001 compared to the WT.* Pak1, p21-activated kinase; PDHA1, pyruvate dehydrogenase E1α subunit.

Taken together, the fact that pyruvate level was increased in MDA-Panc 48 Pak1 KO cells and Pak1 directly phosphorylates PDHA1, led us to explore the influence of Pak1 on PDHA1 catalytic activity. MDA-Panc 48 cells showed significant reduction in phosphorylated PDHA1 levels at 4 μM IPA-3 treatment (Fig. 5*D*), therefore, we attempted to investigate the resulting alteration in PDHA1 activity. Concordantly, IPA-3–treated PDAC cells displayed significantly higher enzymatic activity than the vehicle controls (Fig. 7*A*). Similarly, MDA-Panc 48 Pak1 KO cells exhibited strongly significant increase in PDHA1 activity, which was drastically reduced upon transient transfection with kinase-active Pak1 plasmid. This reduction in the PDHA1 activity upon rescue of Pak1 expression indicated that Pak1 negatively regulates PDHA1 activity (Fig. 7*B*). In order to further validate the regulatory effect of Pak1 on PDHA1 activity, we analyzed the same in stable clones expressing differential levels of Pak1—Pak1 stable rescue and Pak1 overexpression (OE). In the Pak1 stable rescue clone, the restoration of Pak1 expression in Pak1 KO cells led to a moderately significant decline in the PDHA1 activity, comparable with WT cells. The PDHA1 activity of Pak1 OE clones was significantly lower than the control (Fig. 7*C*). In summary, these results indicated a negative regulatory association between Pak1 and PDHA1.

As we have demonstrated that Pak1 phosphorylates PDHA1 at S152, we attempted to decrypt the role of this phosphorylation on the catalytic activity of PDHA1. To study this, we transfected HEK293T cells with PDHA1-WT or PDHA1-S152A plasmids. Upon measurement of PDHA1 enzymatic activity, the cells expressing the PDHA1 phospho-null mutant (S152A) exhibited increased PDHA1 activity compared to those with the PDHA1-WT plasmid (Fig. 7*D*). Additionally, upon transfection with phospho-mimic (S152E) mutant in MDA-Panc 48 Pak1 KO cells, we observed a 2-fold reduction in PDHA1 activity compared to the Pak1 KO cells, showing nearly identical activity to that observed in WT cells (Fig. 7*D*). Thus, it can be inferred that PDHA1-S152E mutant reversed the increase in PDHA1 activity in Pak1 KO cells. These results substantiates that the Pak1 mediated phosphorylation of PDHA1 at S152 negatively regulates its enzymatic activity.

### PDHA1 phosphorylation at S152 alters the glycolytic flux in MDA-Panc 48 cells

**Figure 8:**
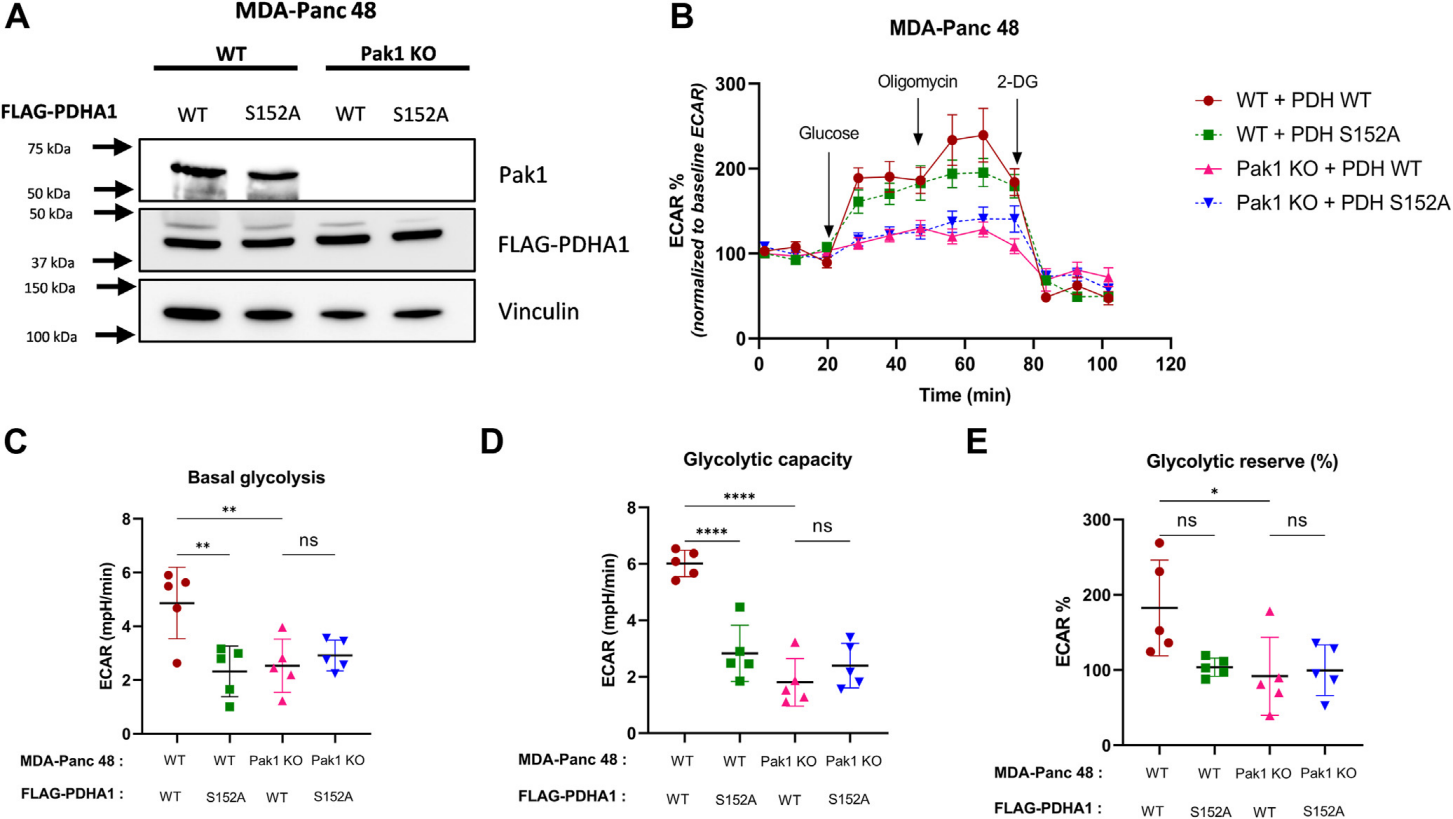
**PDHA1 S152 phosphorylation by Pak1 alters the glycolytic flux in MDA-Panc 48 cells.***A*, Western blot to confirm the transfection of WT and S152 PDHA1 plasmids in Pak1 WT and KO MDA-Panc 48 cells. Assessment of the glycolytic flux of transfected cells using Seahorse extracellular flux (XF96) analyzer. *B*, graphical representation of changes in the extracellular acidification rate (ECAR, mpH/min) upon sequential addition of glucose, oligomycin, and 2-deoxy glucose (2-DG) in MDA-Panc 48 cells; followed by the ECAR values of glycolytic parameters (*C*) basal glycolysis, (*D*) glycolytic capacity, and (*E*) glycolytic reserve % were calculated. Each value represented as mean ± SD (n = 5); (∗*p* < 0.05, ∗∗*p* < 0.005, and ∗∗∗*p* < 0.0001). Pak1, p21-activated kinase; PDHA1, pyruvate dehydrogenase E1α subunit.

Earlier in this study, it was shown that Pak1 expression affected the glycolytic flux in MDA-Panc 48 cells using WT and Pak1 KO cells (Fig. 2, *A*–*D*). To investigate if this decreased glycolytic flux in the absence of Pak1 was by its inhibitory phosphorylation of PDHA1, we transfected MDA-Panc 48 WT and KO cells with PDHA1 WT and PDHA1 S152A phospho-null mutant plasmids (Fig. 8*A*) and followed by Seahorse XF glycolytic stress assay. The assay measures the ECAR, an indirect measure of lactate production, and subsequently indicates glycolysis. As expected, the Pak1 WT/PDHA1 WT cells had higher ECAR under glucose deprivation, which further increased with the injection of saturating concentration of glucose, than Pak1 WT/PDHA1 S152A, and Pak1 KO cells (Fig. 8*B*). These cells also showed 2-fold increase in the basal glycolytic rate (*p* = 0.0036) and 3-fold increase in the glycolytic capacity (*p* < 0.0001) when compared to Pak1 WT/PDHA1 S152A (Fig. 8, *C* and *D*). Reduced glycolytic capacity in the PDHA1 phospho-null mutants indicated the inability of these cells to efficiently utilize glycolysis even when mitochondrial ATP synthesis was inhibited. While the glycolytic reserve followed a similar trend, we did not observe statistical relevance (Fig. 8*E*).

In Pak1 KO MDA-Panc 48 cells, irrespective of PDHA1 WT or S152A transfection, we observed significantly reduced basal glycolysis, maximal glycolytic capacity, and glycolytic reserve, when compared to Pak1 WT/PDHA1 WT cells and equivalent to the WT/PDHA1 S152A cells. Thus, we conclude that diminution of PDHA1 enzymatic activity in the MDA-Panc 48 cells as a result of Pak1-mediated phosphorylation at S152, results in a decrease in the conversion of pyruvate to acetyl-CoA. This leads to an accumulation of pyruvate, which is converted to lactate by LDHA, and a subsequent increase in the ECAR. Further, the comparable results in Pak1 WT/PDHA1 S152A cells and Pak1 KO cells with either PDHA1 WT or S152A mutant indicated specific effects of PDHA1 S152 phosphorylation by Pak1 on glycolysis in MDA-Panc 48 cells. However, it is noteworthy to understand that there might be other pathways involved in the differential glycolytic flux between Pak1 WT and KO MDA-Panc 48 cells and further studies are warranted to identify the same.

## Discussion

Recent studies have consistently demonstrated Pak1 OE in pancreatic cancer, highlighting its involvement in promoting cell proliferation, migration, invasion, and resistance to chemotherapy (9, 14). Though there is substantial evidence on the role of Pak1 in promoting tumorigenesis by regulating the cellular proliferative pathways, its involvement in altering the tumor cell metabolism is not much explored (6). Our study provides insights into the regulation of tumor cellular metabolism by Pak1, thereby promoting tumorigenesis.

In general, tumor cells display increased glucose uptake and glycolysis rate. The findings from our study show significant reduction in the basal glycolytic activity of the PDAC cells upon *Pak1* gene deletion. Consistent with the earlier report, the involvement of Pak1 in regulating glycolysis is independent of KRAS oncogenic mutation as Pak1 KO cells of both MDA-Panc 48 (Mut KRAS) and BxPC3 (WT KRAS) have reduced glycolysis (15). Furthermore, we noticed an increase in the basal respiration level of the MDA-Panc 48 Pak1 KO cells as compared to WT, which nearly reached the maximum respiration rate. The Pak1 KO cells are operating at the maximal respiratory capacity under basal conditions in an attempt to compensate for the glycolytic loss. Also, increased proton leak associated with no significant difference in ATP-linked respiration in Pak1 KO cells indicates reduced mitochondrial coupling efficiency. It highlights that Pak1 gene deletion makes the cells metabolically weak with less reserve capacity to sustain in metabolically challenging conditions. Previous reports have shown that Pak1 serves an essential role in maintaining mitochondrial function, including mitochondrial dynamics, bioenergetics, and apoptosis (16). Research findings indicate that BxPC3 cells exhibit a pronounced glycolytic phenotype and diminished reliance on mitochondrial function, accompanied by aberrant mitochondrial morphology (17). We speculate that the diminished response of BxPC3 Pak1 KO cells to mitochondrial inhibitors may result from a combination of their inherent impaired mitochondrial function and the absence of the Pak1 gene expression. Unlike MDA-Panc 48 cells, the BxPC3 Pak1 KO cells may be incapable of redirecting the metabolic burden toward mitochondrial respiration. Therefore, we preferred to continue the study with MDA-Panc 48 cells.

In Koranova *et al.* 2022, the study explored the Pak1 and Pak2 role in regulating tumor cell metabolism in adherent and leukaemia cells using different approaches—inhibitors, siRNA, and gene KO. The pharmacological inhibition of Pak1/Pak2 and silencing *Pak1/Pak2* using siRNA reduced the tumor cell metabolism—reduced ECAR and OCR. On the contrary, the *Pak1* gene deletion did not show similar impact on the metabolic pathways, increased ECAR and decreased OCR, which we suppose due to the retained weak Pak expression in the KO clones, possibly affecting the study outcome (18). Our study underscores the significant role of Pak1 in orchestrating the energy requirements of the rapidly proliferating cells. We have also explored the impact of Pak1 specifically in the pancreatic cancer cell metabolism. We have performed global metabolomic analysis which revealed pyruvate metabolism as one of the highly dysregulated pathways upon *Pak1* gene deletion. Though Koranova *et al.* have extensively studied the role of Pak1 in glucose homeostasis, ours is the first study to establish the link between Pak1 and pyruvate metabolism. To strengthen our understanding, we have mechanistically studied the regulatory role of Pak1 on PDHA1, thus impacting pyruvate metabolism. Our study mainly focuses on the kinase activity of Pak1, while Koranova *et al.* have explicitly postulated the observed effect as nonkinase function of group 1 Pak.

In order to gain a deeper understanding of the role of Pak1 in tumor cell metabolism, untargeted metabolomic analysis was carried out. Global metabolic profiling recognized pyruvate metabolism as the top enriched pathway modulated by Pak1. Prior research has convincingly demonstrated that pyruvate metabolism serves as a central hub in carbon metabolic pathways. Notably, normal and cancer cells exhibit distinct metabolic signatures in pyruvate utilization, contributing to the metabolic adaptability of cancer cells (19). From our experimental data, Pak1 KO cells had lower pyruvate and higher acetyl-CoA levels. PDHA1, gatekeeper enzyme linking glycolysis to oxidative phosphorylation, mediates the conversion of pyruvate to acetyl-CoA, hence we were inquisitive about the link between Pak1 and PDHA1. Our findings reveal that Pak1 interacts with PDHA1 under physiological conditions, whereas *in vitro* kinase assay shows that Pak1 phosphorylates PDHA1. Pak1 phosphorylation of PDHA1 was confirmed by both radioactive labeling and Phos-tag, followed by pharmacological and genetic perturbation of Pak1 activity/levels. In addition to the *in-vitro* kinase assay with purified PDHA1 protein, Phos-tag analysis of cell lysates gives a definitive proof that PDHA1 is a physiological substrate of Pak1. PDHA1 is predominantly regulated by phosphorylation, impacting its localization, molecular interaction, activity, and stability (20, 21). In our study, we unraveled a novel PDHA1 phosphorylation at serine 152 by Pak1, which was found to inhibit PDHA1 catalytic activity.

Reports have shown that a reduction in PDHA1 expression or activity is associated with unfavorable outcomes in multiple cancer types (22, 23, 24, 25, 26). PDHA1 was also found to play a role in the oncogene induced senescence, which was inhibited by the OE of PDK1 (27). PDHA1 reduction drastically shifts the metabolism from oxidative phosphorylation to lactate production in cancers such as prostate and esophageal squamous cell carcinoma (28, 29, 30). On the other hand, OE of PDHA1 suppressed the glucose uptake and lactate production in hepatocellular carcinoma cells, indicating reduced aerobic glycolysis (31). Consistent with the reports, Pak1 KO cells exhibiting high PDHA1 activity showed reduced glycolysis and increased mitochondrial respiration, favoring oxidative phosphorylation. Additionally, we explored the specific effects of PDHA1 S152 phosphorylation by Pak1 on glycolytic flux. It was observed that the glycolytic flux and other glycolytic parameters were higher in the Pak1 WT/PDHA1 WT cells than Pak1 WT/PDHA1 S152A, Pak1 KO/PDHA1 WT, and Pak1 KO/PDHA1 S152A cells. Previous reports with PDHA1 KO esophageal cancer model showed enhanced glycolytic rate corroborating our hypothesis of PDHA1 S152A mutants with higher enzymatic activity (29).

Given that elevated LDHA levels have been observed in various types of cancer, including PDAC, and are associated with unfavorable prognosis and reduced patient survival (32, 33, 34, 35, 36, 37). Interestingly, the expression of Pak1 positively impacts the activity of the enzyme lactate dehydrogenase (LDHA) (Fig. 7*F*). The LDHA has a crucial role in Warburg effect, catalyzing the conversion of pyruvate to lactate, the final step of anaerobic glycolysis. These observations open a new, wide arena for research on the role of Pak1 in regulating LDHA in tumor cell metabolism.

In summary, the involvement of Pak1 in tumor cell metabolism represents a complex and multifaceted research area, as global metabolomic data analysis reveals that Pak1 regulates crucial metabolic pathways, including glycolysis and various amino acid metabolism pathways. Deleting Pak1 gene expression leads to reduced glycolysis, increased basal respiration, and reduced reserve capacity, consequently affecting the metabolic adaptability of PDAC cells. Pak1 directly phosphorylates PDHA1 at Ser152, inhibiting its activity and thus promoting glycolysis. Additionally, the Pak1 regulates LDHA expression, influencing the Warburg effect by promoting glycolysis. These findings collectively indicate that the Pak1 oncogene promotes a metabolic shift toward aerobic glycolysis, favoring the conversion of pyruvate to lactate rather than being directed to OXPHOS. The findings discussed in this paper shed light on the intricate mechanisms by which Pak1 influences aerobic glycolysis by regulating pyruvate metabolism in PDAC cells. Overall, our study underscores the critical role of Pak1 in metabolic reprogramming and its potential as a therapeutic target in PDAC.

## Experimental procedures

### Cell lines, plasmid, and reagents

The PDAC cell line, MDA-Panc 48, was gifted by Dr Paul J Chiao (University of Texas, M. D. Anderson Cancer Center). BxPC3 cell line CRL-1687 was purchased from the American Tissue Culture Collection. MDA-Panc 48 and BxPC3 were cultured using Dulbecco's modified Eagle's medium with high glucose and RPMI-1640, respectively, supplemented with 10% fetal bovine serum (FBS) and 100 units/ml of ampicillin-streptomycin with amphotericin. The cell culture media and antibiotics were procured from Himedia, with FBS sourced from Gibco. CRISPR/Cas9 KO plasmid specific to human Pak1 (sc-400857-KO-2) were purchased from Santa Cruz Biotechnology. Primary antibodies were procured from Cell Signaling Technologies: Pak1 (2602); PDHA1 (3205); LDHA (3582); and DDK-tag (14793S). The anti-pPak1 (Thr^212^) mAb (3237) was purchased from Sigma. Vinculin antibody (V9131) was purchased from Sigma. Secondary antibodies, horseradish peroxidase-conjugated anti-mouse and anti-rabbit antibodies, were purchased from Rockland Immunochemicals, Inc. Full-length recombinant human pyruvate dehydrogenase PDHA1 protein was purchased from Abcam (ab125602). The lambda protein phosphatase was purchased from NEB (P0753S). pCMV6M-Pak1-T423E plasmid (constitutively active Pak1) was procured from Addgene (Plasmid# 12208). The hPDHA1 plasmid with Myc-DDK-tag (RC201831) was purchased from OriGene Technologies Inc. The QuikChange II site–directed mutagenesis kit (200524) was purchased from Agilent. The primers for generating site-directed mutagenesis are as follows: For phospho-null mutant: FP - 5′-CATACATGTGCATCGCTCCTCCTTTCCCTTTAGC-3′, RP - 5′-GCTAAAGGGAAAGGAGGAGCGATGCACATGTATG-3′; for phosphor-mimic mutant: FP - 5′-CTTGGCATACATGTGCATCTCTCCTCCTTTCCCTTTAGCA-3′, RP - 5′-TGCTAAAGGGAAAGGAGGAGAGATGCACATGTATGCCAAG-3′. The reaction was set for generating site-directed mutations as per the manufacturer’s instructions and the presence of mutation was confirmed by sequencing.

### IHC analysis

The pancreatic cancer TMA PA807 was procured from Biomax as a formalin-fixed paraffin-embedded section. First, the TMA was deparaffinized using xylene and, constitutively, rehydrated with alcohol. Further, peroxide blocking and antigen retrieval was carried out using heated TRIS-EDTA (pH 9.2). The slides were incubated with anti-rabbit Pak1 (dilution ratio 1:100) overnight at 4 °C. The slides were washed, incubated with secondary antibody followed by counterstaining with hematoxylin, and mounted using dibutylphthalate polystyrene xylene mountant solution for imaging and scoring purposes. Images of the tissue section were visualized using the Leica DM 2000 LED light microscope. The Microscope software platform Leica Application Suite was used to capture the IHC images at different magnifications. The slides were used to measure Pak1 expression by the Q-score method (Q = P × I, where P is the percentage of positive cells and I is the intensity of the staining).

### Generation of stable Pak1 KO clone

Stable Pak1 KO clones were generated by transfecting the PDAC WT cells with CRISPR-Cas9 Pak1 KO plasmids using Fugene HD, as the transfection agent (1:3). After 72 h, the transfected cells were sorted as a single cell based on the GFP expression using BD FACS Aria III Cell Sorter. The single cells grew into colonies, and subsequently, the colonies formed were screened using Western blot analysis to confirm the presence of Pak1 KO clone.

### Generation of stable Pak1 OE clones and Pak1 stable rescue clones

Stable Pak1 OE clones and Pak1 stable rescue clones were established by transducing the retroviral particles in PDAC WT and Pak1 KO cells, respectively. HEK293T cells were transfected with either pBABE-puro control or pBABE-Pak1 plasmid along with pUMVC, VSV-G plasmid, and the supernatant containing retroviral particles was collected after 48 h of transfection. The retroviral particles were added to the PDAC cells for transduction, followed by puromycin selection. The OE of Pak1 was confirmed through immunoblot analysis.

### Clonogenic survival assay

Cells (250 cells per plate) were seeded in a 60 mm dish and allowed to form individual colonies by maintaining at 37 °C in the incubator for 2 weeks. The colonies were fixed using a fixative solution (30% v/v methanol and 10% v/v acetic acid) and stained using the crystal violet solution (0.5% w/v crystal violet prepared in 50% v/v methanol). The number of colonies formed was counted manually, and subsequently, the survival fraction of Pak1 KO was calculated relative to the WT.

### Wound healing assay

Cells were seeded to reach 90% confluency in a 60 mm dish. Once the monolayer was attained, overnight serum starvation was given followed by mitomycin treatment (5 μg/ml) for 2 h. A scratch was made using a pipette tip (p20) and the detached cells were removed by PBS wash, followed by media change. Images were taken at different time points using a phase contrast microscope (Carl Zeiss microscope) to calculate the percentage of wound closure.

### Migration assay

Cells (20,000 cells/well) resuspended in media (without FBS) were added to the upper chamber. Complete media with serum was added to the lower chamber and the cells were allowed to migrate for 24 h. The migrated cells were fixed, stained using Hemocolor stain, and further imaged using a Carl Zeiss microscope to count the number of cells migrated.

### Real-time metabolic analysis using Seahorse flux analyzer

Seahorse XF24 Extracellular flux analyzer (Agilent Technologies) provides a real-time assessment of the bioenergetic profile of the live cells by directly measuring the ECAR (release of proton) as well as the OCR. All the consumables were purchased from Agilent Technologies. Cells were seeded in a XF24 cell culture plate at a seeding density of 8 × 10^4^ cells/well and 6 × 10^4^ cells/well for MDA-Panc 48 and BxPC3, respectively. For the glycolysis stress test, the cells were incubated with the XF base medium supplemented with 2 mM glutamine in a non-CO_2_ incubator for 1 h at 37 °C, before the start of the assay. The baseline ECAR value was then measured, followed by the sequential addition of glucose (10 mM), oligomycin (ATP synthase inhibitor, 1 μM for MDA-Panc 48, and 1.5 μM for BxPC3), and 2-deoxyglucose (50 mM). The glycolytic parameters were computed as per the manufacturer’s instructions (Cat. No 103020-100).

For the Mito stress test, the cells were incubated with XF base medium supplemented with 10 mM glucose, 1 mM pyruvate, and 2 mM glutamine in a non-CO_2_ incubator for 1 h at 37 °C, before the assay. The OCR was measured under basal conditions, followed by the stepwise addition of the inhibitors: oligomycin, carbonyl cyanide-4 (trifluoromethoxy) phenylhydrazone (uncoupler) used at the concentration of 0.5 μM for MDA-Panc 48 and 0.25 μM for BxPC3, followed by rotenone/antimycin A (complex I and III inhibitors) at the concentration of 0.5 μM each. The mitochondrial parameters were computed as per the manufacturer's instructions (Cat. No 103010-100).

To study the effect of PDHA1 phosphorylation on glycolytic flux, similar protocol was adopted to the 96-well method using Seahorse XF96 Extracellular flux analyzer (Agilent Technologies). Pak1 WT and KO MDA-Panc 48 cells were each transfected with PDHA1 WT or PDHA1 S152A plasmid (2.5 μg each). After 24 h, the cells were trypsinized and 1 × 10^4^ cells/well were seeded into Seahorse XF96 cell culture microplate (Agilent) and incubated at 37 °C and 5% CO_2_ overnight. On the day of the test, the cells were changed to Assay media (Himedia AT063A) and incubated in non-CO_2_ incubator at 37 °C for not more than an hour prior to the run. The injection ports were loaded with glucose, oligomycin, and 2-deoxyglucose and the run was carried out as per the manufacturer’s instructions. Data was analyzed using Agilent Wave Pro software (https://www.agilent.com/en/product/cell-analysis/real-time-cell-metabolic-analysis/xf-software/seahorse-wave-pro-software-2007523) and graphs were generated in GraphPad Prism 5 (https://www.graphpad.com/support/prism-5-updates/), and significance was determined by one-way ANOVA followed by Tukey’s multiple comparisons test.

### Western blotting

Total cellular protein was extracted from cells by lysing with radioimmuno precipitation assay (RIPA) buffer (150 mM NaCl, 50 mM Tris–HCl, 1 mM EDTA, 1% Nonidet P-40, 0.25% sodium deoxycholate) with 1× protease inhibitor cocktail (purchased from Roche). The protein content was quantified using Bio-Rad DC protein estimation reagents as given in the manufacturer’s protocol. The protein samples were subjected to SDS-PAGE electrophoresis and then transferred onto the nitrocellulose membrane. The membrane was blocked with 5% nonfat milk solution (prepared in 1× Tris buffered saline 0.1% Tween-20) at room temperature for 1 h and incubated overnight with primary antibody at recommended dilution (1:1000) at 4 °C. Later, the membrane was washed and incubated with an appropriate horseradish peroxidase-conjugated secondary antibody (dilution ratio of 1:5000). The chemiluminescent signal resulting from protein–antibody interaction was enhanced by using a Bio-Rad enhanced chemiluminescent reagent kit and visualized by ImageLab software (https://www.bio-rad.com/en-in/product/image-lab-software?ID=KRE6P5E8Z). The housekeeping gene was also probed for normalizing the protein loaded.

### Metabolite extraction and LC-MS/MS analysis

Cells at 80% confluency were used for metabolite extraction. The cells were collected using trypsin and washed with ice-cold PBS (1×) thrice. Further metabolism was stopped by quenching the cells with liquid nitrogen for 5 min and stored at −80 °C till metabolite extraction. The cells were resuspended in 1 ml of 50 mM triethyl ammonium bicarbonate buffer, sonicated (30 s ON and 5 s OFF at 30% amplitude for 2 min) followed by centrifugation at 10,000*g* for 5 min. The collected supernatant was used for metabolite extraction as well as for protein normalization. The metabolite extraction was performed with a combination of solvents acetonitrile: methanol: Water taken in the ratio 2:2:1. The sample with extraction solvent was vortexed thoroughly, followed by sonication for 15 min at room temperature. After sonication, the sample was centrifuged at 10,000*g* for 15 min at 4 °C. The supernatant was collected, filtered using a 0.22 μm filter, and vacuum-dried using Speedvac for LC-MS/MS analysis. Analysis of the metabolite extract was carried out using a QTRAP 6500 mass spectrometer (ABSciex) coupled with Agilent 1290 infinity II liquid chromatography system with a C18 RRHD Zorbax column (20 × 150 mm, 1.8 μm particle size). Data acquisition was performed with Analyst software version 1.6.3 (https://sciex.com/products/software/analyst-software), which includes an analyst device driver, for defining the analysis parameters. The metabolite separation was performed using liquid chromatography for precisely 25 min, with solvent A (0.1% v/v formic acid, prepared in MS grade water) and solvent B (0.1% v/v formic acid in 90% v/v acetonitrile) at a consistent flow rate of 0.25 ml/min. The gradient flow for liquid chromatography (LC) was set as 2% B for 1 to 10 min, 30% B for 10 to 14 min, 60% B for 14 to 18 min, 95% B for 18 to 21 min, and 2% B for 21 to 25 min. The mass spectrometry data was acquired in the information-dependent acquisition (low mass mode) method, with inbuilt enhanced mass spectra (EMS) to enhanced product ion modes. The EMS mode, used for untargeted metabolomics, scans for a maximum number of metabolites in the sample. Collision-induced dissociation (CID), a high-energy collision was used to carry forward the top five spectra identified in EMS mode to enhanced product ion (MS2) mode. Furthermore, the metabolite data was obtained at both positive and negative polarities operated at 4500 V and −4500 V, respectively, with a probe temperature of 450 °C. The declustering potential of 100 V and the collision energy of 40 V were used. The data were acquired from four biological replicates for each group. The output files in. wiff format were used for further processing.

### Metabolite identification and data analysis using metaboanalyst

The output files from Analyst software (.wiff format) were converted to .mzML format using the Proteowizard MSconvert tool. The metabolite data processing was carried out using MS-dial software (version 4.9.22; https://systemsomicslab.github.io/compms/msdial/main.html) in centroid mode. The peak detection, MS2 deconvolution, peak identification, and peak alignment parameters were all set to default, except the accurate *m/z* tolerance value (0.05 Da for MS1 and 0.5 Da for MS2). Untargeted metabolites identification in the complex mixture was done in MS-dial using the spectral libraries (both positive and negative) available in .msp format. The resultant file containing the alignment ID, *m/z* value, retention time, and peak area was converted to .csv format for further analysis. The data normalization and statistical analysis (*t* test) were done using Metaboanalyst 6.0 (www.metaboanalyst.ca). The FC of the metabolites in Pak1 KO relative to WT was calculated. The functional enrichment analysis with the list of DAMs (0.75 ≤ FC ≥ 1.3) was performed to study the impact of Pak1 in altering the metabolic profile of PDAC cells.

### *In vitro* kinase assay

*In vitro* kinase assay was set with glutathione-*S*-transferase–tagged Pak1 (Kinase) and recombinant human PDHA1 (2 μg, substrate) protein in kinase assay buffer with radiolabeled γ32-ATP at 37 °C for 30 min. Likewise, the control reactions were set up for validating the Pak1 phosphorylation activity by using myelin basic protein as positive control and esterase as negative control. The reaction mixture was denatured, loaded onto 12% SDS-PAGE, and transferred onto the nitrocellulose membrane. The blot was visualized by Ponceau-S stain and exposed to the storage phosphor screen overnight. The phosphor screen was scanned by using a Phosphoimager (Bio-Rad, GS-525, QuantityOne software; https://www.bio-rad.com/en-in/product/quantity-one-1-d-analysis-software?ID=1de9eb3a-1eb5-4edb-82d2-68b91bf360fb or GE Healthcare, Typhoon FLA 9500, Imagequant TL software; https://info.cytivalifesciences.com/image-analysis-software.html). The assay was also repeated with experimental conditions, with and without Pak1 to check whether the observed PDHA1 band was due to the autophosphorylation effect.

The Phos-tag acrylamide solution (5 mM) was purchased from Wako Chemicals (Cat. No: 304-93526). The Phos-tag acrylamide preferentially binds to and separates different phosphoproteo forms from unphosphorylated protein. *In vitro* kinase assay was set up with recombinant hPDHA1 protein with and without Pak1 as described above. The recombinant hPDHA1 protein simultaneously treated with lambda protein phosphatase as per the manufacturer’s protocol served as unphosphorylated control. Similarly, *in vivo* phosphorylation was confirmed with whole cell lysates. The whole-cell lysates were collected with EDTA-free RIPA buffer. The samples were denatured, subjected to SDS-PAGE with 20 μM Phos-tag acrylamide and 40 μM MnCl_2_, and then transferred onto nitrocellulose membrane for further analysis.

### Phosphosite identification by LC-MS/MS

The sites phosphorylated on PDHA1 by PAK1 were analyzed by LC-MS/MS, following in-gel trypsin digestion of *in vitro* kinase assay products. The peptides were separated using Dionex UltiMate 3000 RSLC nano System (Thermo Fisher Scientific) interfaced with an Orbitrap Exploris 240 Hybrid Mass Spectrometer (Thermo Fisher Scientific). The peptides were eluted in a step gradient of 1 to 50% solvent B (0.1% formic acid [FA] in 80% acetonitrile) at a flow rate of 350 nl/min over 20 min. A survey full scan MS (from *m/z* 375–1200) was acquired in time-dependent acquisition mode in the Orbitrap with a resolution of 120,000. Peptides with charges 2 to 6 were considered for MS acquisition and the peptides were then selected in a data-dependent manner for MS2 scan events using a Quadrupole filter. Peptides were fragmented using higher energy collisional dissociation with 30% normalized collision energy and the resolution for MS2 was set to 15,000. The mass spectrometry–derived data was analyzed using SEQUEST HT, MS Amanda 3.0, and COMET search algorithms through Proteome Discoverer platform (https://www.thermofisher.com/in/en/home/industrial/mass-spectrometry/liquid-chromatography-mass-spectrometry-lc-ms/lc-ms-software/multi-omics-data-analysis/proteome-discoverer-software.html?erpType=Global_E1%2CSAP_PR1_2040) (version 3.1., Thermo Fisher Scientific). Search parameters including maximum of two missed cleavages, propionamide on cysteine was set as static modification, while oxidation of methionine and phosphorylation of serine and threonine as dynamic modifications. Target decoy PSM validator was used to validate results with strict target false discovery rate set to 0.01 and relaxed target rate set to 0.05. iptmRS was used to calculate phosphorylation site probabilities.

### Coimmunoprecipitation assay

Lysates from MDA-Panc 48 WT and Pak1 KO were collected using NP-40 buffer. MDA-Panc 48 Pak1 KO lysate was used as the control to detect the nonspecific binding of the antibody. Lysates (500 μg) were precleared with 20 μl of A/G beads for 1 h followed by overnight incubation with Pak1 antibody at 4 °C. The following day, the 40 μl of A/G beads was added to the mixture and kept in the rotor for 4 h at 4 °C. The beads were washed with ice-cold RIPA buffer and eluted samples were denatured with 2× loading dye by boiling at 95 °C for 5 min. The denatured sample was then centrifuged at 12000*g* for 5 min, and the supernatant was loaded onto 10% SDS-PAGE. Ten percent of the lysates were used as the input.

### Enzyme activity assays

The pyruvate dehydrogenase (PDH) activity of MDA-Panc 48 WT and Pak1 KO was measured using the PDH assay kit purchased from Sigma (Cat No. MAK-183). The lactate dehydrogenase (LDHA) activity was measured using the LDH assay kit purchased from Abcam (Cat No. Ab102586). The assays were performed in triplicates with 1 μg of the protein lysate as per the manufacturer’s instructions. Both PDHA1 and LDHA activity of WT and Pak1 KO cells were reported as nmol min^−1^ mg^−1^.

### Statistical analysis

Data represented as mean ± SD was derived from the biological replicates. Student *t* test was performed to analyze the significance between the groups using GraphPad Prism 9 software. A *p* value of ≤0.05 is considered to be statistically significant.

## Data availability

All the data available is presented in the MS and is available with the Corresponding Author on reasonable request.

## Supporting information

This article contains supporting information.

## Conflict of interest

The authors declare that they have no conflicts of interest with the contents of this article.
