# Supplementary material for: Pak1 dysregulates pyruvate metabolism in PDAC cells by exerting a phosphorylation-mediated regulatory effect on PDHA1
Source: J Biol Chem. 2025 Mar 14;301(4):108409. doi: 10.1016/j.jbc.2025.108409 (PMC12013493; doi:10.1016/j.jbc.2025.108409)
Supplement: Supplementary Figure S1 [file mmc1.docx]

**Supplementary Figure**

Supplementary figure S1:

**b**


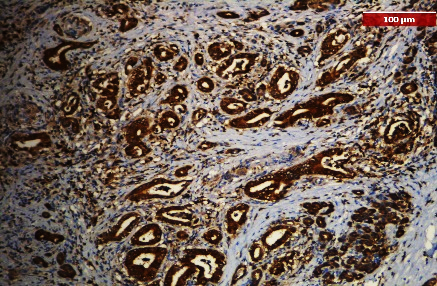

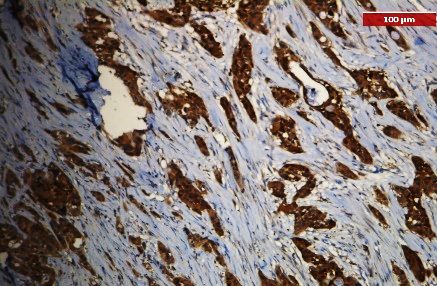

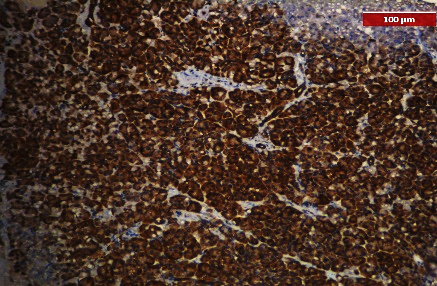

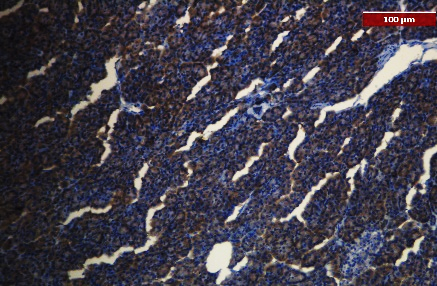


**PDAC tissue**

**Normal tissue**

**a**

1. Representative image of IHC staining of Pak1 in Tissue microarray (TMA) with tumor tissue and its adjacent normal pancreatic tissue samples (10X magnification).
2. Graphical representation of the mean Q-score of Pak1 staining calculated in normal and PDAC tissue samples (n = 40). *(***p < 0.0005 compared with normal tissue).*
